# Incidence of depression and anxiety among working men and women: Evidence from a cross-sectional survey in Indian call centers

**DOI:** 10.1371/journal.pmen.0000460

**Published:** 2025-11-03

**Authors:** Srishti Goel, Rishiraj Bhagawati, Dong Zhou, Jamie Mullins, Sarojini Hirshleifer, Deepshikha Batheja

**Affiliations:** 1 Economics, One Health Trust, Bangalore, Karnataka, India; 2 Development Studies, One Health Trust, Bangalore, Karnataka, India; 3 Department of Cultural Industry and Management, Shanghai Jiao Tong University, Shanghai, China; 4 Department of Resource Economics, University of Massachusetts Amherst, Amherst, Massachusetts, United States of America; 5 Economics Department, University of California, Riverside, California, United States of America; 6 Max Institute of Healthcare Management, Indian School of Business, Hyderabad, Telangana, India; PLOS: Public Library of Science, UNITED KINGDOM OF GREAT BRITAIN AND NORTHERN IRELAND

## Abstract

Employee mental well-being, supported by a positive work environment is crucial to enhancing firm productivity and sustaining long-term growth. However, research on the mental well-being of working populations in developing countries remains limited. This study examines the mental health status and key predictors of depression and anxiety in working men and women in India, one of the world’s most populous countries. For this, we collected quantitative data from 2,698 individuals using self-administered online surveys. We used bivariate and multivariate regressions to analyze the predictors of depression and anxiety in working men and women. We also used Ordinary Least Squares (OLS) method to assess the association between gender and depression and anxiety. Finally, we assessed the role of personal stressors, work stressors, use of social media and siblings to understand the gender differences in anxiety and depression using OLS regression methods. Around 23% of female and 17% of male workers in our sample reported symptoms of either depression or anxiety, or both. Key predictors of mental health issues of the working population in the call centers included younger age, financial difficulties and, conflicts with supervisors, with additional mental health predictors of experience of domestic violence, unmarried status, and lack of siblings for women. Our regression results suggest that women in our sample exhibited 0.13 standard deviations higher depression and 0.26 standard deviations higher anxiety than men. Furthermore, women experiencing high personal and work-related stress, along with frequent social media use, reported the highest levels of depression and anxiety. Lastly, we find that relative to men without siblings, men with siblings report lower depression levels, and women without siblings report higher anxiety. These insights underscore the need for targeted interventions to support the mental health of working populations, particularly among women in developing contexts.

## 1. Introduction

Mental health disorders are a major public health concern worldwide, with significant consequences for individuals and economies. In 2017, approximately 14% of the Indian population experienced mental disorders of varying severity [1]. This includes 45.7 million individuals with depressive disorders and 44.9 million with anxiety disorders, with women showing a notably higher prevalence than men. These statistics highlight a substantial mental health burden in the world’s most populous country. While awareness and resource for mental health are improving in metropolitan cities in India, it still remains substantially poor in other semi-urban and rural areas [2]. Young adults, particularly during the COVID-19 pandemic, faced heightened risks of depression, anxiety, and stress [3]. Research also indicates that women are disproportionately affected by mental health disorders compared to men, with women being twice as likely to develop depression over the course of their lives [4]. Beyond biological factors, socioeconomic and cultural pressures further exacerbate mental health risks for women. In particular, working women may face increased vulnerability to mental illness due to the added burden of unpaid care work, driven by adverse gender norms in these regions. Therefore, studying the mental health of young workers in semi-urban regions, particularly women, is crucial to understanding and addressing these disparities.

Assessing the mental health of the working population and fostering safe work environments is essential for maintaining a productive workforce. Several factors contribute to increased levels of depression and anxiety among young working adults, including work-related stress [5], cultural beliefs, societal expectations [6], and socio-economic challenges [7]. Additionally, issues such as domestic violence and family expectations can further deteriorate the women’s mental health [8]. This study is grounded in two key theoretical frameworks. The Job Demand-Control Model explains how high job demands and low control over work environments contribute to stress and mental health outcomes, particularly in toxic and male-dominated workplaces [9]. Feminist labor market theories suggest that women’s experiences in the workforce are influenced by structural inequalities, gender norms, and unpaid care work, which disproportionately burden them compared to men [10].

Among various occupational groups, call center employees across the globe face high risks of mental health problems, including anxiety, depression, and burnout, due to high job demands, emotional labor, and constant performance monitoring [11–14]. Studies from countries such as Italy, South Korea, and the Philippines have highlighted how the nature of call center work, especially the emotional toll of handling distressed or aggressive customers, contributes to psychological distress. These findings underline the call center industry as a high-stress work environment with international relevance.

The existing literature on mental well-being of working women in India primarily relies on qualitative methods with small sample size [15–17]. It is especially pertinent to understand the mental well-being of working women, especially in the context where gender plays a significant role. Our study expands on existing knowledge by estimating the prevalence of self-reported anxiety and depression among working women in India compared to men. We also examine the socio-economic, personal, and work-related factors that predict these mental health outcomes. Furthermore, we explore how gender influences mental well-being and interacts with stressors both at home and in the workplace, with a focus on urban areas where most employment opportunities are concentrated.

This study explores three key research questions concerning the mental well-being of India’s working population. The first question investigates the various socio-economic, personal, and work-related factors that predict the prevalence of self-reported anxiety and depression among working men and women in India. The second question examines the influence of gender on the prevalence of self-reported anxiety and depression within the working population. Finally, we also explore the relationship between interaction of gender with stressors on the prevalence of self-reported anxiety and depression. By addressing these questions, this study provides new insights into the intersection of gender, employment, and mental well-being in India, with implications for policy and workplace reforms.

We used primary data collected from a quantitative survey of 1,565 men and 1,133 women working in call centers across five Indian cities. The surveys evaluated the mental health outcomes of working professionals and their associations with factors such as job satisfaction, support from supervisors and colleagues, prospects of marriage, having sick or unwell parents, and domestic violence, among others. We conducted descriptive analysis and, bivariate and multivariate regression analysis to study this first question on predictors of mental health of men and women. Additionally, we performed ordinary least squares (OLS)/linear probability model regressions to study the last two research questions on whether gender is associated with mental well-being.

This research contributes to three major strands of literature. First, it expands on studies examining the inverse relationship between mental illness and labor force participation [18–21]. Mental illnesses negatively impact worker productivity, leading to substantial economic costs for firms [22]. Next, we contribute to the literature on higher prevalence of anxiety and depressive disorders in females than in males [1,23,24]. Gender differences in mental health outcomes have been associated with various factors, including sexual abuse, intimate partner violence, stress around childbirth, and adverse socio-cultural norms [25–28]. Third, we contribute to the growing literature on constraints to women participating in the workforce. While much of this literature has focused on supply-side constraints to female labor force participation in developing countries, particularly in India [29,30], this paper also examines the demand-side factors which constrain women’s employment such as stressful and toxic male-dominated work environments.

Moreover, several gender-related shifts in daily routines were observed during the COVID-19 pandemic. For instance, women in India experienced an increased burden of domestic chores, along with a greater reduction in time spent with friends compared to men [31]. Improving the mental well-being of the population in this large nation may be crucial in improving labour market outcomes for both men and women [32–34].

## 2. Materials and methods

### 2.1. Ethics statement

Informed consent was obtained from all study participants through an online consent form. Participants could only proceed with the survey if they explicitly agreed to the consent form. Only individuals above the age of 18 were eligible to participate. Data confidentiality was strictly maintained, with all survey responses de-identified and securely stored in a cloud system accessible only to the research team. All the survey participants were provided with the contact details of a leading tele-mental health support organization in India, in case they required assistance following the completion of the survey. The study received IRB approval from Ashoka University (IRB Reference: 28_21_Dasgupta), and no consent waivers were granted.

### 2.2. Data

In this study, we adopted a quantitative research design, utilizing self-administered online surveys. The sample was gathered through a combination of convenience and voluntary response sampling methods. We opted for convenience sampling as one of the co-authors had previously conducted a study in this call center, facilitating easier access to participants and streamlining the recruitment process. Participants were recruited with the assistance of the call center’s human resources (HR) personnel, who facilitated the distribution of online consent forms. HR personnel were only involved in distributing the consent forms and had no access to individual responses, ensuring participant confidentiality and minimizing response bias. All employees were encouraged to participate, and those who provided consent proceeded to complete the survey. This approach was chosen to maximize participation and ensure broad representation across the workforce. To reduce potential sampling bias, we ensured that employees from various job roles, including customer support representatives, team leaders, and managers, were encouraged to participate. Additionally, we aimed for balanced participation across gender and experience levels. Employees from different shifts were invited, and the survey remained open for a flexible period to accommodate varying work schedules. To acknowledge their time and effort, participants were compensated upon survey completion. The final dataset includes 1,565 men and 1,133 women from India, all employed in the call center industry across five cities. The survey was conducted between July and August 2022.

We focused on the call center industry as it is one of the largest private sector employers in India, with a young workforce that is particularly susceptible to mental health challenges due to the high-pressure nature of the work and frequent interactions with difficult customers. To facilitate data collection, we collaborated with a call center company and surveyed their employees across five locations in four Indian states: Udaipur (Rajasthan), Bengaluru and Hubli (Karnataka), Bhopal (Madhya Pradesh), and Jamshedpur (Jharkhand). Participants were compensated for the time spent completing the survey.

The survey covered a range of topics, including self-reported levels of depression and anxiety, as well as demographic information such as age, education, work experience, and marital status. Questions related to stress sources, both personal and professional, were informed by qualitative interviews and existing mental health literature. The qualitative interviews, which were conducted in the pilot phases of the study, included in-depth discussions with 10 call center employees and management personnel across the study sites. These interviews helped shape the study design and stressors that were explored through the quantitative surveys. Personal stressors included pressures related to marriage, parental health, relationship breakups, domestic violence, financial problems, family restrictions, and societal stigma surrounding low-paid call center jobs in India. Workplace stressors were grouped into categories addressing job dissatisfaction, organizational challenges, supervisory issues, and coworker relationships. These factors were assessed using indices constructed through a variance-weighted method, as outlined by Anderson, 2008 [35]. Once the quantitative survey was finalized, the call center management was asked to review the survey tools to ensure readability and relevance of the questions.

#### 2.2.1. Measuring the mental health of employees.

Our primary outcome variable in this study is mental health, assessed using two established scales: the Patient Health Questionnaire (PHQ-9) for measuring depression and the Generalized Anxiety Disorder (GAD-7) scale for anxiety^1^. These scales have been validated for the Indian setting. In addition, we conducted preliminary qualitative interviews with call center employees to vet the language of the survey [36]. The PHQ-9 scale ranges from 0 to 27, with scores categorized as follows: 0–4 for minimal depression, 5–9 for mild, 10–14 for moderate, 15–19 for moderately severe, and 20+ for severe depression [37]. Similarly, the GAD-7 scale ranges from 0 to 21, with scores of 0–4 indicating minimal anxiety, 5–9 mild, 10–14 moderate, and 15 + severe anxiety [38].

We define two mental health indicators for each participant. The first is a binary variable, coded as 1 if the individual reports symptoms indicative of depression or anxiety, using a threshold of 10 or higher on the PHQ-9 and GAD-7 scales [37,39]. The second measure is a continuous variable capturing symptom severity, derived through standardized indices. To calculate this, we convert each survey item into z-scores by subtracting the mean and dividing by the standard deviation of the control group (working men).

#### 2.2.2. Summary statistics.

Table 1 presents the descriptive statistics for all the call center employees surveyed in our sample. Almost 17% women and 13% men reported being moderately or severely depressed, and 17% women and 12% men reported being moderately or severely anxious in our sample. Overall, 23% of the women and 17% of the men in our sample reported symptoms of either depression or anxiety or both. Women reported significantly higher depression and anxiety than their male counterparts in India. In terms of job roles, 90% of women are in customer service positions versus 85% of men, while 18% of men hold senior positions compared to 11% of women (both p < 0.01). Social media use is more common among men (64% vs. 52%, p < 0.01). Additionally, 86% of women live with parents versus 75% of men (p < 0.01), and 93% of women have siblings compared to 89% of men (p < 0.01). Relationship break-ups affect 2% of women and 4% of men (p < 0.01), and men report higher organizational (p < 0.01) and management constraints (p < 0.01) as work stressors. The sample surveyed was relatively young, with almost 80% women and 75% men being in the 18–25 age group. Approximately half our sample had atleast completed high school while the other half had at least completed university level of education.

**Table 1:** Summary statistics of the outcome variables.

Variables	Female (n = 1133)		Male (n = 1565)		P-value
	Mean (1)	Standard Deviation (2)	Mean (3)	Standard Deviation (4)	Mean difference (col 1- col 3)
Self- Reported Anxiety (continous)	0.25	1.10	0.00	1.00	<0.01
Self- Reported Anxiety (0/1)	0.17	0.38	0.12	0.32	<0.01
Self- Reported Depression (continous)	0.13	1.02	0.00	1.00	0.01
Self- Reported Depression (0/1)	0.17	0.38	0.13	0.34	0.01
Self- Reported Depression and/or Anxiety	0.23	0.42	0.17	0.38	<0.01
Customer service executive	0.90	0.30	0.85	0.36	<0.01
Senior position in the organization	0.11	0.31	0.18	0.38	<0.01
Use social media	0.52	0.50	0.64	0.48	<0.01
Religious majority (Hindu)	0.71	0.45	0.73	0.44	0.23
Live with parents	0.86	0.35	0.75	0.43	<0.01
Married	0.15	0.36	0.15	0.36	0.93
Have siblings	0.93	0.26	0.89	0.31	<0.01
Lost anybody close in COVID-19	0.19	0.39	0.20	0.40	0.57
Personal Stressors	0.43	0.49	0.46	0.49	0.14
Pressure of getting married	0.05	0.22	0.06	0.24	0.42
Sick parents	0.06	0.24	0.06	0.24	0.68
Break-up with boyfriend/girlfriend	0.02	0.13	0.04	0.20	<0.01
Financial problems	0.27	0.45	0.30	0.46	0.22
Domestic violence	0.02	0.15	0.03	0.16	0.54
Family/ movement restrictions	-0.02	0.93	0.00	1.00	0.63
Pressure to leave the current job	-0.14	0.63	0.00	1.00	<0.01
*Work Stressors*	0.36	0.47	0.38	0.48	0.13
Nature of employment	-0.05	0.92	0.00	1.00	0.17
Organizational constraints	-0.13	0.68	0.00	1.00	<0.01
Management constraints	-0.09	0.77	0.00	1.00	<0.01
Co-workers	-0.06	0.74	0.00	1.00	0.12
**Demographic Variables**	**N**	**Percentage**	**N**	**Percentage**	
Age group					
18-25	892	79%	1,152	74%	
26-30	148	13%	250	16%	
31-40	85	8%	141	9%	
Above 40	8	1%	22	1%	
Education					
Secondary school or less	10	1%	35	2%	
Completed High School	532	47%	797	51%	
Completed university or higher	591	52%	733	47%	

### 2.3. Methodology

First, we perform both bivariate and multivariate regression analyses to examine the predictors of depression and anxiety among working men and women in India separately. Bivariate analyses are conducted to identify significant associations between the dependent and independent variables. Independent variables that demonstrate a significant association with the dependent variables at a p-value of less than 0.05 are then included in the multivariate model.

Second, in order to study the association of gender on mental health in the workplace, we estimated the following Ordinary Least Squares (OLS) regression model:


$${\text{y}}_{\text{ij}}\;=\;\alpha \;+\;\beta {\text{Female}}_{\text{ij}}+\;\gamma {\text{Z}}_{\text{ij}}\;+\;{\text{u}}_{\text{j}}+\;\epsilon_{\text{i}}$$ (1)

In this analysis, y_ij_ represents a continuous variable indicating the level of depression or anxiety for individual ‘i’ residing in city ‘j’. The levels of anxiety or depression have been normalized as an index for better comparability. The variable Female_ij_ is binary, taking a value of 1 if the individual is female and 0 if male. The coefficient β captures the average effect of gender on the mental health status of working individuals. The vector Z_ij_ includes individual control variables such as age, religion, job status, and marital status. Additionally, city fixed effects (u_j_) are included to account for correlations in mental health outcomes within cities, recognizing that socio-cultural and environmental factors—such as weather—can influence mood.

Next, we assess the role of personal stressors, work stressors, use of social media and siblings to understand the gender differences in the mental health outcomes of anxiety and depression by estimating the following OLS regression specification.


$${\text{y}}_{\text{ij}}\;=\;\alpha \;+\;\beta_{\text{1}}{\text{Female}}_{\text{ij}}\;\;*{\left(\text{1}-\;\text{interaction}\right)}_{\text{ij}}+\beta_{\text{2}}{\text{Male}}_{\text{ij}}\;\;*{\left(\text{interaction}\right)}_{\text{ij}}+\;\beta_{\text{3}}{\text{Female}}_{\text{ij}}\;\;*{\left(\text{interaction}\right)}_{\text{ij}}+\gamma {\text{Z}}_{\text{ij}}\;+\;{\text{u}}_{\text{j}}+\;\epsilon_{\text{i}}$$

y_ij_ represents a continuous variable which indicates the level of depression or anxiety for individual ‘i’ in city ‘j’. The interaction term includes binary variables of having any personal stress, having any work stressors, using social media and having siblings. The term {Female_ij_*(1 − interaction)_ij_} captures the effect of being female when the interaction condition is absent, while {Male_ij_*(interaction)_ij_} reflects the effect of being male when the interaction is present. The term {Female_ij_*(interaction)_ij_} allows for assessing how the relationship of being female changes in the presence of the interaction. The vector Z_ij_ includes individual control variables such as age, religion, job status, and marital status, u_j_ accounts for unobserved city-level factors, and ∊_i_ captures individual-level random error, together enabling a nuanced exploration of how personal stressors or other relevant factors affect mental health differently by gender.

To assess potential multicollinearity in the regression models that included control variables, we computed Variance Inflation Factors (VIFs) for each specification (see S1 and S2 Tables). VIF values exceeding the commonly accepted threshold of 10 indicated problematic collinearity. As a result, we excluded education level from the final models to improve estimation precision and model stability. All data, analysis code, and research materials are accessible. Quantitative data analysis was conducted using Stata 14. This study’s design and analytical approach were not preregistered.

## 3. Results

We first conducted bivariate regressions for self-reported symptoms of anxiety and depression with all the personal and work stressors and, demographic variables. The significant variables were from the bivariate regression were included in the multivariate analysis. These variables included age, education, use of social media, being married, pressure to get married, having sick parents, financial problems at home, break up with partner, domestic violence at home, family restrictions, pressure to leave current job, nature of work, organizational-related stressors, issue with supervisors and co-works, having siblings, being Hindu, being a customer service executive, senior position in the organization and losing somebody during COVID-19.

**Fig 1:**
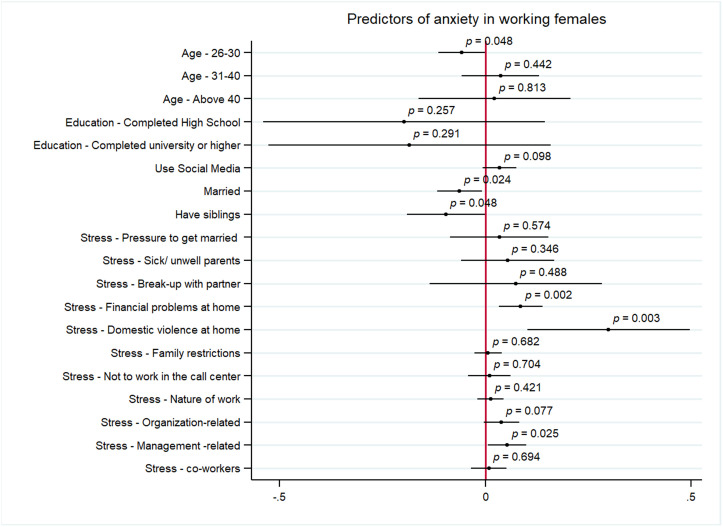
Predictors of symptoms of anxiety among women.

**Fig 2:**
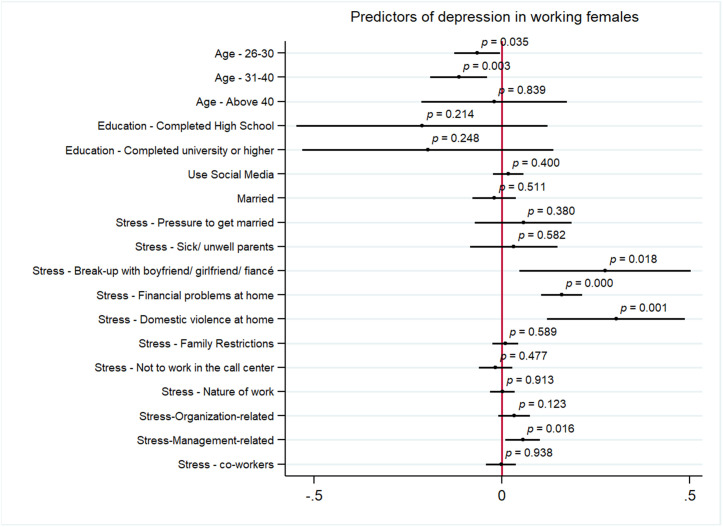
Predictors of symptoms of depression among women.

Fig 1 and Fig 2 represent the results from the multivariate analysis assessing the predictors of symptoms of anxiety and depression among women, respectively. The significant predictors of symptoms of anxiety among working women included personal stressors of financial problems at home (AOR: 0.08, 95% CI 0.03 to 0.13, p < 0.05), domestic violence at home (AOR: 0.30, 95% CI 0.10 to 0.49, p < 0.05) and work-related stressor of issues with supervisor (AOR: 0.05, 95% CI 0.00 to 0.01, p < 0.05). Age of 26–30 years (AOR: -0.05, 95% CI -0.11 to 0, p < 0.05), being married (AOR: -0.06, 95% CI -0.11 to 0.00, p < 0.05) and having siblings (AOR: -0.09, 95% CI -0.19 to 0.00, p < 0.05) were negatively correlated with symptoms of anxiety for women. The predictors of depression for working women included, personal stressors of break-up with partner (AOR: 0.27, 95% CI 0.04 to 0.50, p < 0.05), financial problems at home (AOR:0.16, 95% CI 0.10 to 0.21, p < 0.05), and domestic violence at home (AOR: 0.30, 95% CI 0.12 to 0.48, p < 0.05), and work stressor of issues with supervisor (AOR: 0.05, 95% CI 0.01 to 0.10, p < 0.05). Being of age 26–30 (AOR:0.06, 95% CI -0.12 to 0.00, p < 0.05) and 31–40 years (AOR: 0.11 95% CI -0.18 to -0.03, p < 0.05) is negatively correlated with symptoms of depression among women.

**Fig 3:**
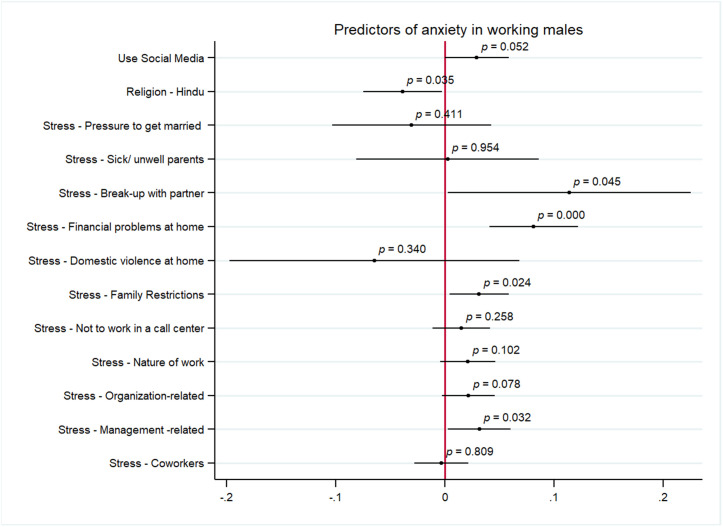
Predictors of symptoms of anxiety among men.

**Fig 4:**
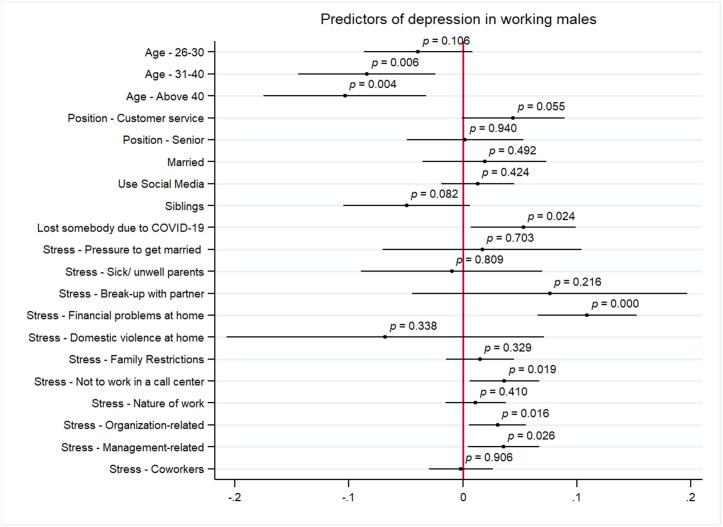
Predictors of symptoms of depression among men.

Fig 3 and Fig 4 represent the results from the multivariate analysis assessing the predictors of symptoms of anxiety and depression among men, respectively. The significant predictors of anxiety were personal stressors of break up with partner (AOR:0.11, 95% CI 0.00 to 0.22, p < 0.05), financial problems at home (AOR: 0.08, 95% CI 0.04 to 0.12, p < 0.05) and family restrictions (AOR: 0.03, 95% CI 0.00 to 0.05, p < 0.05). Issues with supervisor was the only significant work stressor positively correlated with anxiety for men (AOR: 0.03, 95% CI 0.00 to 0.06, p < 0.05). Additionally, being Hindu was negatively correlated with anxiety (AOR:0.03 95% CI -0.07 to 0.00, p < 0.05) and using social media was positively related with anxiety for men (AOR: 0.02, 95% CI 0.00 to 0.05, p < 0.05). Being of age 31–40 (AOR:0.08, 95% CI -0.14 to -0.02, p < 0.05) and above 40 years (AOR: 0.10, 95% CI -0.17 to -0.03, p < 0.05) is negatively correlated with symptoms of depression among men. Other predictors of depression among men include personal stressors of financial problems at home (AOR: 0.11, 95% CI 0.06 to 0.15, p < 0.05) and pressure to leave current job (AOR: 0.03 95% CI 0.00 to 0.06, p < 0.05), and work stressors include organizational constraints (AOR: 0.03, 95% CI 0.00 to 0.05, p < 0.05) and issues with supervisor (AOR:0.03, 95% CI 0.00 to 0.06, p < 0.05). Finally, we also find that losing someone in COVID-19 pandemic was a significant predictor of anxiety in men (AOR:0.05 (AOR: 0.03, 95% CI 0.00 to 0.09, p < 0.05).

Table 2 examines the effect of gender on depression and anxiety levels among working individuals in India, using continuous measures for each condition to capture more nuanced differences. Our analysis reveals that working women report depression levels that are 0.13 standard deviations (SD) higher than their male counterparts. This gender difference in depression persists even after accounting for potential confounding factors such as education level, religion, job status, and marital status, with women still exhibiting a 0.13 SD higher depression level.

**Table 2:** Association of gender on anxiety and depression in India.

	(1)	(2)	(3)	(4)
VARIABLES	Self- Reported Depression (without controls)	Self- Reported Depression (with controls)	Self- Reported Anxiety (without controls)	Self- Reported Anxiety (with controls)
Female	0.13***	0.13***	0.25***	0.26***
	(0.04)	(0.04)	(0.04)	(0.04)
Controls	No	Yes	No	Yes
Observations	2,698	2,698	2,698	2,698
Adjusted R^2^	0.00	0.02	0.01	0.02

Note: All regressions include city fixed effects. We have controlled age, religion, job level and marital status. Coefficients significantly different from zero at 99% (***), 95% (**) and 90% (*) confidence level.

Similarly, the data indicate that working women experience anxiety at levels approximately 0.25 SD higher than men. When we control for education, religion, job level, and marital status, the gap in reported anxiety levels slightly increases, with women showing 0.26 SD higher anxiety than men. These findings suggest that gender-based disparities in mental health outcomes remain substantial even after adjusting for socioeconomic and demographic variables.

Table 3 summarizes the effects of gender, in combination with various stressors—namely, personal stress, work stress, social media use, and the presence of siblings—on continuous measures of anxiety and depression in India. The results reveal distinct patterns across gender and stressor types, with certain groups exhibiting significantly higher mental health impacts. We find substantial gender differences in the association between personal stress and mental health outcomes. Compared to men without personal stress, men experiencing personal stress report depression levels 0.69 SD higher, while women without personal stress show only a modest increase of 0.14 SD. In contrast, women with personal stress exhibit the highest increase, reporting depression levels 0.84 SD above those of men without personal stress. A similar trend is observed for anxiety: men with personal stress report 0.68 SD higher levels, women without personal stress report 0.25 SD higher, and women with personal stress show the largest increase at 0.99 SD. This pattern highlights that women experiencing personal stress consistently report the highest levels of both anxiety and depression, underscoring the intensified relationship of personal stress on women’s mental health.

**Table 3:** Relationship of gender with other stressors on anxiety and depression in India.

	(1)	(2)	(3)	(4)	(5)	(6)	(7)	(8)
VARIABLES	Self-Reported Depression	Self-Reported Depression	Self-Reported Depression	Self-Reported Depression	Self-Reported Anxiety	Self-Reported Anxiety	Self-Reported Anxiety	Self-Reported Anxiety
Male*Personal stress	0.69***				0.68***			
	(0.05)				(0.05)			
Female* No Personal stress	0.14***				0.25***			
	(0.04)				(0.05)			
Female* Personal stress	0.84***				0.99***			
	(0.06)				(0.06)			
Male*Work stress		0.75***				0.77***		
		(0.05)				(0.05)		
Female* No Work stress		0.17***				0.29***		
		(0.04)				(0.05)		
Female* Work stress		0.86***				1.03***		
		(0.06)				(0.06)		
Male*Use social media			0.13**				0.15***	
			(0.05)				(0.05)	
Female* Do not use social media			0.10*				0.20***	
			(0.06)				(0.06)	
Female* Use social media			0.31***				0.50***	
			(0.06)				(0.06)	
Male*Have siblings				-0.23**				-0.10
				(0.10)				(0.09)
Female*Do not have siblings				0.17				0.51***
				(0.17)				(0.17)
Female* Have siblings				-0.10				0.15
				(0.10)				(0.09)
Mean								
Observations	2,698	2,698	2,698	2,698	2,698	2,698	2,698	2,698
R-squared	0.14	0.14	0.03	0.03	0.13	0.14	0.04	0.03

Note: All regressions include city fixed effects. We have controlled for age, religion, job level and marital status. Coefficients significantly different from zero at 99% (***), 95% (**) and 90% (*) confidence level.

Examining work-related stress reveals comparable gender-specific differences. Men with work stress report depression scores 0.75 SD higher than men without work stress, whereas women without work stress show a modest 0.17 SD increase. However, women with work stress report 0.86 SD higher depression relative to men without work stress, indicating a stronger effect of work stress on depression in women. For anxiety, men with work stress report scores 0.77 SD higher than men without work stress; women without work stress report 0.29 SD higher, and women with work stress show the highest increase at 1.03 SD. These findings suggest that work stress has a particularly pronounced influence on women’s anxiety and depression, similar to the pattern observed with personal stress.

Gender differences also emerge in the relationship between social media use and mental health. Compared to men who do not use social media, men who do report depression and anxiety levels 0.13 SD and 0.15 SD higher, respectively. For women, those who do not use social media report increases of 0.1 SD in depression and 0.2 SD in anxiety, while women who use social media report substantially higher increases of 0.31 SD in depression and 0.5 SD in anxiety. These findings suggest that social media use is associated with greater anxiety and depression in women than in men, with the largest association observed among women who engage with social media. Lastly, we assess the association between sibling presence and self-reported anxiety and depression. Men with siblings report depression scores 0.23 SD lower than men without siblings, suggesting a possible protective effect of siblings on men’s mental health. In contrast, women without siblings report anxiety levels 0.51 SD higher than men without siblings, indicating that the absence of siblings may be associated with higher anxiety among women.

## 4. Discussion

This paper addressed three key questions regarding the mental well-being of India’s working population: (a) the socio-economic, personal, and work-related predictors of depression and anxiety among the workforce, (b) the association between gender and these mental health outcomes, and (c) relationship between the interaction of gender with stressors and the prevalence of self-reported anxiety and depression. Our first set of findings indicate that self-reported depression and anxiety among working women in India are associated with factors such as young age, domestic violence, financial difficulties, and work-related issues with supervisors. Additionally, depression is linked to relationship problems, while anxiety is related to being unmarried and lacking siblings. Among working men, depression and anxiety are also associated with financial problems and conflicts with supervisors, with depression further linked to young age and family pressure against their current employment (due to stigma around low-paying call center jobs). Anxiety in men is tied to personal issues, such as relationship challenges and family or mobility restrictions. Our second research objective finds that working women in India exhibit higher depression and anxiety than men. Finally, our third set of findings indicate that women with personal stress, work stress and social media use have the highest levels of depression and anxiety. We also find that men with siblings report lower levels of depression and women without siblings report higher levels of anxiety.

The findings of this study highlight the critical need to prioritize the mental well-being of the workforce from an employment policy perspective. Mental illnesses take a toll on worker productivity, resulting in substantial economic costs for both firms and employees [10]. Our findings indicate that there is a high incidence of depression and anxiety among the working population that requires urgent attention. Existing literature also discusses the gender differences in mental health outcomes being linked with a range of factors including sexual abuse, intimate partner violence, stress around childbirth, and adverse social and cultural norms [4,14–16]. This study also finds evidence on the role of adverse gender norms on women’s mental health in India, with domestic violence being significantly correlated with depression and anxiety among working women in India, but not among working men. Being unmarried is also significantly associated with anxiety for this subgroup.

Most of the literature has focused on supply-side constraints to female labor force participation in developing countries, particularly in India [17,18]. Apart from studying supply-aide factors, our paper also studies demand-side factors which could be constraining women’s labor supply such as stressful, toxic or male-dominated environments. We contribute to this literature by providing evidence that even financially independent women are more prone to depression and anxiety than men in India. This indicates that taking care of the mental health needs of the working women in India will be crucial to retain them in the work force.

These findings are consistent with the Job Demand-Control Model, which posits that mental health outcomes worsen in settings characterized by high demands and low autonomy. This is particularly evident in the significant role that work-related stressors, such as issues with supervisors, play in predicting anxiety and depression for both men and women. Additionally, the results support insights from feminist labor market theory, which frames women’s labor force experiences as shaped by intersecting structural constraints, including gendered power relations, social expectations, and the dual burden of paid and unpaid work. The higher prevalence of mental health issues among women, even when financially independent, underscores the persistent influence of unpaid care responsibilities, societal expectations around marriage and family, and exposure to gendered risks such as domestic violence.

Implementing appropriate workplace policies can help alleviate certain stressors. For example, stress related to heavy workloads, challenging interactions with colleagues and supervisors, and pressures to compete with peers could be significantly reduced through transparent grievance processes. Training managers in communication and conflict resolution, as well as providing allowances for professional upskilling programs, are additional measures that could prove effective. Furthermore, workplace policies should aim to enhance gender equality by promoting strategies such as fair decision-making and educating employees on social norms, which can help reduce biases that hinder gender equality [40].

Our findings also suggest a potential protective role of sibling relationships in shaping mental well-being, particularly for men. Men with siblings reported lower levels of depression, while women without siblings were more likely to experience anxiety. This may reflect the buffering effects of emotional or logistical support that siblings can offer, especially in navigating family expectations, stigma, or life transitions. For women, the absence of such support may amplify stress in both professional and personal domains, particularly in settings where unmarried or financially independent women face social scrutiny. While this finding warrants further exploration, it highlights the importance of informal support systems in shaping gendered mental health outcomes in the workforce.

Among the personal stressors identified, concerns about the health of family members—reported by several respondents—could be alleviated if workplaces provided family health insurance and offered flexible work arrangements to accommodate personal responsibilities. Other personal stressors highlighted in this study could be mitigated by promoting psychotherapy and counselling for employees, especially in the ongoing recovery from the effects of the COVID-19 pandemic [41]. Encouraging open discussions on mental health in the workplace, de-stigmatizing the practice of seeking mental health support, and improving access to psychotherapy services would enable employees to prioritize their mental health and better cope with the daily stressors of both work and personal life. Of these, improving access to mental health resources is particularly critical in today’s environment. Employers can address this by offering employee assistance programs, partnering with mental health providers, or providing access to telemedicine services. Additionally, the study finds that adverse gender norms play a significant role in women’s mental health in India, with domestic violence strongly correlated with depression and anxiety among working women, but not men. This finding has important policy implications, underscoring the urgent need to address women’s mental health.

Potential long-term impacts of these stressors, which remain outside the scope of this paper, could extend beyond individual well-being, potentially influencing economic mobility and workforce participation rates of the region. Persistent mental health challenges lead to lower job retention and diminished professional growth, particularly among women who already face structural barriers in the labor market, and may contribute to the high attrition rate that the call center industry usually experiences. Moreover, untreated mental health conditions may exacerbate economic inequalities by limiting access to stable employment and reducing overall productivity of the workforce. This highlights the need for sustained interventions that not only address immediate workplace stressors but also build long-term mental health resilience among employees across sectors, ensuring their continued participation in the workforce and broader economic development.

Beyond its immediate findings, this study contributes by highlighting mental health disparities within a rapidly expanding labor sector that remains underrepresented in global occupational health research. The gender-focused lens adopted here is both timely and essential, offering important insights for designing workplace mental health interventions in India and comparable contexts. The study also provides a useful framework for future research in other high-strain, low-regulation sectors where similar vulnerabilities may exist.

## 5. Limitations

This study provides a valuable contribution to mental health and women’s work literature. However, this study is not one without limitations. Our main limitation is that the study is based on self-reported symptoms of depression and anxiety rather than a clinical diagnosis by a healthcare professional. Nevertheless these self-reported measurement scales are validated and widely used in the literature [26]. While the lack of preregistration raises concerns about analytical flexibility, we followed best practices, including predefined hypotheses and robustness checks, to ensure reliability. The second limitation of the study is that our list of personal and work stressors used in the survey may not have accounted for all the stressors faced by these workers and this list may not be exhaustive. However, these stressors were derived from the existing literature and some qualitative interviews and discussions with the call center employees prior to the quantitative survey.

Another shortcoming of this study is that it focuses exclusively on the business process outsourcing (BPO) sector in urban areas across tier-, tier-2 and tier 3 cities in India, where significant concentration of BPO operations is located [42], rather than examining a broader range of industries. While this narrow focus limits the generalizability of our findings to other sectors, it is important to note that the BPO industry is one of the largest and fastest-growing employment sectors in India, with over 4.1 million workers [43]. Additionally, the BPO industry is expanding rapidly in other developing countries, such as the Philippines and China, making it a relevant sector for studying workplace mental health [44,45]. Further, any results in this study are correlational, as the absence of baseline mental health measures may limit causal interpretation. To deepen understanding of causal pathways and the evolution of workers’ mental health outcomes, subsequent empirical research could employ longitudinal designs alongside in-depth qualitative interviews. These approaches would offer richer insights into how specific stressors interact with workplace environments and individual circumstances to influence mental health trajectories. In addition, future work can focus on building causal evidence on the impact of psychotherapy training in the workplace on employee well-being and productivity, with the use of clinical measures of mental disorders.

## 6. Conclusion

Examining the mental health of working men and women is essential, as our findings reveal notably high levels of anxiety and depression within this population. Approximately 20% of our sample reported symptoms of depression or anxiety, which is well above the national average of 14%. This highlights the urgent need for targeted mental health support in the workplace. This study provides novel insights into the prevalence of mental illness among working individuals in India, one of the world’s most populous countries, and identifies key factors contributing to poor mental health. Compromised mental health not only diminishes quality of life but also leads to economic losses and decreased productivity. These findings underscore the importance of dedicating more attention and resources to address the mental health challenges faced by the working population in India. Strengthening workplace mental health policies could also play a vital role in reversing India’s declining female labor force participation, a trend observed over the last two decades.

A stronger institutional response is required beyond the existing voluntary programs. Policymakers should prioritize integrating mental health programs into existing occupational health frameworks, ensuring access to counselling services, recurring stress management training programs, and flexible work arrangements This should be accompanied by statutory measures such as stress audits, mandatory mental health days, and legal protections against discrimination for employees experiencing mental health conditions. Future research in this area should also explore the long-term impact of workplace mental health interventions, with a focus on sector- and location-specific challenges and gender disparities in mental health outcomes. Further studies could also examine the role of gender-specific employer attitudes, social stigma, and the effectiveness legal protections in shaping mental health outcomes among India’s workforce.

## Supporting information

S1 Table VIFs for association of gender on anxiety and depression in India. (XLSX)

S2 Table VIFs models assessing gender and stressor interactions in relation to anxiety and depression. (XLSX)

S1 Data Codes. (DO)

S2 Data Data. (DTA)
